# Evaluation of the effect of the gold nanoparticles prepared by green ‎chemistry on ‎the ‏treatment of cutaneous candidiasis

**DOI:** 10.18502/cmm.7.1.6176

**Published:** 2021-03

**Authors:** Hassan Ayad Kareem, Hayder Mahmood Samaka, Wasna’a Mohamed Abdulridha

**Affiliations:** 1 Department of Microbiology, Faculty of Veterinary Medicine, University of Kufa, Al-Najaf, Iraq; 2 Department of Basic Sciences, College of Dentistry, University of Kufa, Al-Najaf, Iraq

**Keywords:** *Candida albicans*, Cutaneous candidiasis, Gold nanoparticles, Olive leaves

## Abstract

**Background and Purpose:**

Mineral nanoparticle synthesis via green chemistry is ‎considered a novel ‏procedure ‎that ‎has been introduced into some ‏‎‎industries and medical fields. This ‎paper aimed to focus on ‏synthesized gold ‎nanoparticles ‎‎‎‎(‎AuNPs‎) prepared via green chemistry and ‎their usage in the ‏treatment of cutaneous ‎candidiasis.‎‎

**Materials and Methods:**

This study was performed on the green synthesis of AuNPs using olive leaf extract as a reducing ‎agent‎. The ‎UV‎ visible spectroscopy, X-ray diffraction, and atomic force microscopy techniques ‎were used to detect ‏the concentration of the prepared AuNPs‎. ‎The agar gel diffusion method was used to test ‏the ‎antifungal activity of the ‎‎prepared AuNPs *in vitro*. ‏Antifungal efficacy of the AuNPs *in vivo* ‎was tested by the ‎induction of‎‎ cutaneous ‎candidiasis in mice‎.‎This research was conducted on four groups of mice‎. Groups 1 and 2 were used to evaluate the effectiveness of the AuNPs suspension ‎and ‏Nystatin ointment in the treatment ‎of clinical infection, respectively. Groups 3 ‎and ‎4 were the infected ‎and the non-infected control groups, respectively.‎

**Results:**

Based on the findings, the AuNP synthesis using olive leaves was ‎a suitable and ‎secure method. Moreover, it was found that the AuNPs concentration of 40.77 ng‏\‏ml represented the minimum ‎inhibitory concentration for the ‎inhibition of the *Candida albicans*. The prepared AuNPs were more effective than Nystatin ‎in the ‏treatment ‎of cutaneous candidiasis.‎‎

**Conclusion:**

Preparation of AuNPs via green chemistry using olive leaves as a reducing ‎agent is a ‏safe ‎and easy procedure that can be performed to produce AuNPs. In this study, the AuNPs ‎displayed antifungal ‏activity ‏both *in vitro* and in vivo.‎

## Introduction

Green ‎chemistry is a branch of nanotechnology that is considered safe for humans, animals, ‎plants, and the environment and is widely used as it does not cause ‎environmental pollution [ 1 , 2 ]. Properties of metallic nanoparticles support various applications, such as serving as catalysts and sensing components; moreover, they can be used in optical devices and biomedical applications [ 3 , 4 ]. For example, gold nanoparticles (AuNPs) are used in medical applications since they can penetrate the cell wall of a wide range of microorganisms, such as fungi, and make genetic or metabolic modifications that can kill microorganisms [ 3 ]. 

*Candida albicans* is an opportunistic, dimorphic ‎microorganism that can cause several diseases in humans and animals due to its ability to adapt to severe environmental conditions, despite being a part of the normal ‎microflora in humans and animals [ 5 ]. One of the properties of *Candida* is the ability to ‎cause infection in most parts of ‎the human ‎body‎, such as the respiratory, digestive, and urinary systems, as well as the skin. It is also considered the most important cause of opportunistic ‎mycoses worldwide [ 6 ], can lead to a variety of superficial and systemic ‎infections, and colonize medical devices [ 7 ]. Such characteristics make this microorganism the most serious fungus in terms of hospital infections [ 8 , 9 ].

Various antifungal agents have been used in clinics to eliminate *Candida* infections, such as nystatin, fluconazole, and clotrimazole, which have ‎toxic effects on humans [ 10 ]. For this reason, scientists have resolved to reduce or eliminate ‎the toxic effect of these medications by using alternatives, and nanotechnology is one of the ‎successful methods in this regard. In green chemistry methods, chemical ‎agents do not consist of chemical materials, such as sodium hydroxide (NaOH) or hydrogen chloride (HCL), or other agents that are very harmful to the body or ‎the environment. Therefore, it is now the best way to prepare medications and other substances ‎for treatment [ 10 ]. 

This study aimed to focus on the antifungal effect of AuNPs ‎prepared by green chemistry on the cutaneous candidiasis infections, ‎using albino mice as a model. Several other procedures are used to produce AuNPs include the chemical reduction ‎method, ‎laser ablation, and the sol-gel method. However, these are not recommended due to the alkaloids and acidic substances used in them, which cause side effects in the ‎human ‎body [ 11 ]. Therefore, the green chemistry method is the best and safest way ‎to produce ‎nanoparticles [ 12 ].‎

## Materials and Methods

### Preparation of the olive leaf extract

The fresh olive leaves were washed twice with distilled water and then rinsed with ethanol (70%) to remove their dust and bacteria before cutting them into small pieces. Afterward, 5 g of these pieces were added to 150 ml of distilled water, and this mixture was boiled in a water bath for 15 min. Next, the broth was filtered using filter paper and stored in a refrigerator at 4 oC for later use when needed [ 13 ].

### Preparation of ‎ AuNPs

In typical experiment conditions, 3 ml of 0.02 mM hydrogen tetrachloroaurate (III) (HAuCl_4_.4H_2_O, 99.99%) from Direvo Industrial Biotechnology, Germany, was mixed in a test tube with 1 ml of olive leaf extract and stirred vigorously for 15 min on a heater stirrer at 50 °C. An excellent indication of the synthesis of AuNPs is when the color turns to dark yellow [ 12 , 14 ].

The prepared AuNPs were checked using UV-visible spectroscopy, X-ray diffraction (XRD), and atomic force microscopy (AFM). The UV-visible spectra were investigated over a 400–800 nm range with a UV-1650 PC UV-visible spectrophotometer (Shimadzu Corporation, Japan). The structure of the produced AuNPs was examined via XRD (XRD-6000; Shimadzu Corporation, Japan‎). The XRD patterns were recorded at a scan speed of 4°/min, and the AFM was carried out on a DX-700HS spectrometer (Shimadzu Corporation, Japan). 

### Antifungal activity of the prepared AuNPs‎

The agar well diffusion method was carried out to study the antifungal effects of the prepared AuNPs suspension on a pathogenic strain of *Candida albicans* obtained from a previous study [ 15 ]. An overnight well-grown colony of *C. albicans* was suspended in 1 ml of sterile saline solution (NaCl 0.85%), and the concentration was adjusted to match the 0.5 McFarland turbidity standard (1.4 X 10^6^ CFU\ml) [ 16 ]. The inoculum suspension was spread equally on a Casitone agar plate using a cotton swab, and eight equal wells, whose maximum diameter was 10 mm, were made in the agar plate [ 17 ]. 

Afterward, 50 µl of a two-fold dilution of the prepared AuNPs suspension was distributed in six out of the eight wells on the plate, starting with a concentration of 326.12 ‎ng\ml‎. Well C+, the positive control, received a nystatin suspension in a concentration of ‎102.9 ‎mg\ml and well C-, the negative control, received the olive leaf extract suspension. This test was performed three times before the results were confirmed. It should be mentioned that the inhibition zones were recorded after 24 and 72 h (CLSI document M44-A2) [ 17 ].

### Induction of cutaneous candidiasis

In total, 20 female mice (BALB/c strain) with a weight range of 30-40 g (12-14 weeks old) orally received ‎the immunosuppressive medication, Prednisolone, at a dose of 1 mg\kg for five days ‎to prepare them for the induction of the cutaneous candidiasis [ 18 ]. After five days of pre-infection treatment, the ‎experimental animals were divided randomly into four groups. Groups 1, 2, and 3 were exposed to ‎the cutaneous infection, and group 4 was the non-infected control group. The ‎infection was induced by spreading a massive growth of a pathogenic *C. albicans* strain on ‎the dorsal region of the experimental mice using ‎a cotton-tipped swab. The ‎experimental mice were placed under observation until the manifestation of the clinical ‎lesion [ 19 ].

The mice in group 1 (G1) were treated topically with the crude AuNPs suspension. The mice in group 2 (G2) were considered the control group and ‎treated with Nystatin ointment 100,000 I.U/g (Mycodin^®^, SDI, Iraq), as the drug of ‎choice for the treatment of cutaneous candidiasis. The mice in group 3 (G3) remained untreated as the ‎infected control group.‎

 The lesion score system by Westhoff et al. was ‎used to rank ‎and follow up the ‎responses to treatment [ 20 ], and the Kruskal_Wallis nonparametric one-way ANOVA test was used to statistically analyze the data. The study procedures and protocols were approved by the Animal ‎Ethics Committee at the University of Kufa (code: 774\13-01-2020) ‎.

## Results

### Characterization of the prepared gold ‎nanoparticles

**Figure 1:**
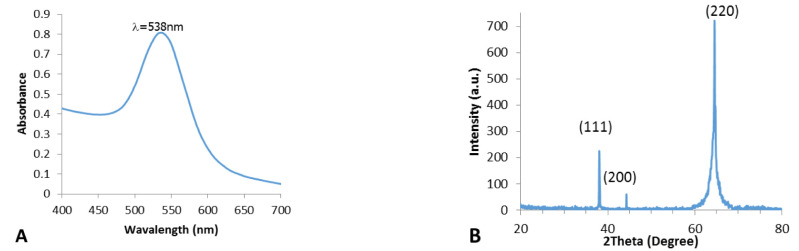
A) The UV–Vis spectrum of AuNPs showing absorption within the wavelength of 538 nm. B) The X-ray diffraction analysis of AuNPs shows peaks at values of 38.1, 44.3, and 64.6 ‎with ‎orientations of 111, 200, and 220, respectively.

For an analytical study of the prepared AuNPs suspension, the amount of absorption within the wavelength of 400–700 nm was observed via a UV Visible spectroscopy. According to Figure 1, A, there is an absorption band at approximately 538 nm due to surface plasmon resonance (SPR) in the AuNPs. The SPR band centered at 538 nm confirms the formation of AuNPs in the solution [ 21 ]. 

The XRD analysis showed peaks at values of 38.1, 44.3, and 64.6, with orientations of 111, 200, and 220, respectively (Figure 1, B), which is typical of the structure of AuNPs. The peak intensity profiles were confirmed through their comparison with data from JCPDS card No. 04-0784. Notable line broadening of the diffraction peaks with low intensity was an indication that the synthesized materials were in the nanometre range. The average particle size was calculated based on the full width at half the maximum of the diffraction peaks, using the Scherrer equation. The average particle size of the AuNPs was found to be 29.16 nm. 

### Calculation of the concentration of AuNPs in a colloidal solution

Concentration of AuNPs was calculated using the following method [ 22 ]

• The average number of atoms per nanoparticle:

$N=\frac{\pi \rho D^{3}}{6M}N_{A}$ (1)

Here, N is the number of atoms per nanoparticle, π=3.14, ρ is the density of gold (19.32 g/cm^3^), D is the average diameter of nanoparticles (29.16 nm), M is the atomic mass of gold (196.96657A g), and N_A_ is Avogadro’s number (6.023×10^23^); therefore, assuming 100% conversion of all Au ions to AuNPs, N is equal to 7665993.

• The molar concentration of the nanoparticles solution:

$C=\frac{N_{T}}{\mathrm{NV}N_{A}}$ (2)

Here, C is the molar concentration of the AuNPs colloidal solution, N_T_ is the total number of Au atoms added as HAuCl_4_.4H_2_O (0.02 mM), N is the number of atoms per nanoparticle which is 1, and V is the volume of the reaction colloidal in L; hence, the concentration is 326.12 ‎ng\ml.

### Antifungal activity of the prepared AuNPs

**Figure 2:**
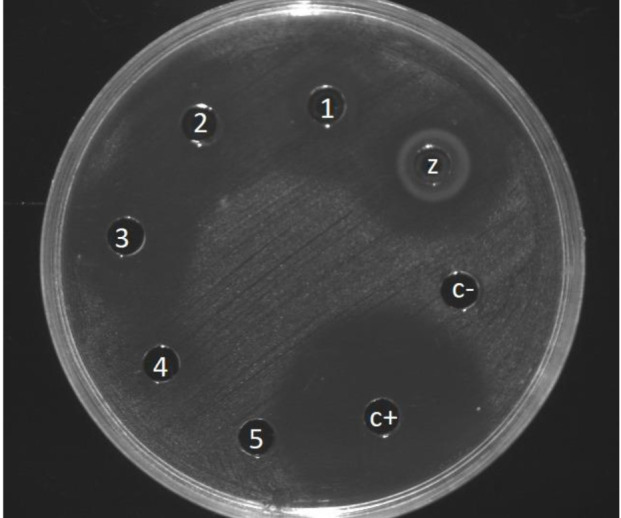
Antifungal activity of serial dilution of AuNPs suspension; concentration of ‎40.77 ‎ng\ml represents the minimum inhibitory concentration (well 4); C+ represents the nystatin suspension as the positive control, and C- represents olive leaf suspension as the negative control.

Based on the outcome of the agar well diffusion, the prepared AuNPs had a clear inhibition effect on *C. albicans* on the plate at the minimum inhibitory concentration of 40.77 ‎ng\ml (Figure 2). Moreover, it was found that the antifungal effect of the AuNPs suspension increased with the increase of the concentration of the AuNPs suspension.

### In vivo experiment

**Figure 3:**
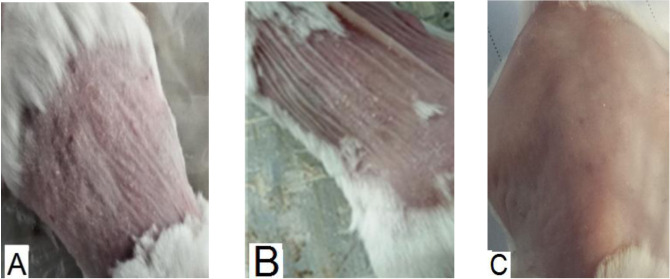
Efficacy of prepared AuNPs suspension in the treatment of cutaneous candidiasis *in vivo*; pictures A, B, and C show ‎the stages of response to treatment by AuNPs suspension.

According to the results of the *in vivo* experiment, the prepared AuNPs suspension had good pharma-cological efficacy in the treatment of cutaneous candidiasis, compared to nystatin (Figure 3). The mice of G1 were entirely cured after three days of treatment with the AuNPs ‎suspension, while it took four ‎days for the mice in G2, which were treated with nystatin, to recover. The treatment efficacy of the AuNPs suspension at a concentration of 326.12 ‎ng\ml was statically significant (P<0.05), compared to nystatin ointment (Table 1).

**Table1:** Results of ranking analysis of study groups show that the group treated by AuNPs has significant differences from the others

Group	Number	Day 1		Day 2		Day 3		Day 4		Day 5	
		Mean	S.D.	Mean	S.D.	Mean	S.D.	Mean	S.D.	Mean	S.D.
G1	5	2^a^	0.71	1.8^a^	0.45	1.2^a^	0.45	1^a^	0.00	1^a^	0
G2	5	2.4^b^	0.55	2.6^b^	0.55	2b	1.00	1.6^b^	0.55	1^a^	0
G3	5	4^c^	0.00	4^c^	0.00	4^c^	0.00	4^c^	0.00	4^c^	0
G4	5	1^d^	0.00	1^d^	0.00	1^d^	0.00	1^a^	0.00	1^a^	0

*The letter “a” in superscript indicates the significant differences (P<0.05) between G1 and ‎G2 during all experiment days.‎

## Discussion

Olive leaf extract is able to reduce tetrachloride gold (III) ions ((-)) to ‎AuNPs in an aqueous solution of hydrogen tetrachloroaurate (III) tetrahydrate (HAuCl_4_·4H_2_O), giving a luminescent emission at 400-700 nm. Concentration of leaf ‎broth may affect ‎luminescent emission of the crude suspension and may interfere with ‎the standard ‎emission of AuNPs [ 23 ].

The AuNPs have mostly been used indirectly as a coupler to a photosensitizer in the treatment of cutaneous fungal infections [ 24 ]. Little data is available on the direct use of AuNPs prepared by green chemistry in the treatment of cutaneous candidiasis. Nevertheless, the results of this study are in line with those of the existing studies which indicated the antifungal effect of AuNPs [ 25 , 26 ]. Moreover, the results of this study were consistent with those of the previous studies which revealed that AuNPs bound to the *Candida* cell wall through electrostatic interactions and released reactive oxygen species that interfered with the signals between cells, caused cell damage, and induced apoptosis [ 3 ]. Besides, in the present research, it was found that this effect increased with the increase of the concentration of AuNPs.

## Conclusion

Olive leaves are good, safe, and cheap materials that can be used as reducing agents in green chemistry to produce ‎AuNPs. Based on the results, the produced AuNPs suspension had a desirable antifungal effect on the plate (*in vitro*) and *in vivo* in the treatment of cutaneous candidiasis‎.‎ Therefore, the production of AuNPs via a green chemistry procedure using olive leaves can be a ‎suitable and safe alternative to other antifungals that are used for the treatment of cutaneous candidiasis.

## Authors’ contribution

S. H.M, A.W.M, and K. H.A. contributed to the design and ‎implementation of the research, analysis of the results, and preparation of the ‎manuscript.‎

## Financial disclosure

This research did not receive any specific grant from funding agencies in the public, commercial, or not-for-profit sectors.
